# Supplementary material for: Imaging nanomagnetism and magnetic phase transitions in atomically thin CrSBr
Source: Nat Commun. 2024 Jul 17;15:6005. doi: 10.1038/s41467-024-49717-9 (PMC11255258; doi:10.1038/s41467-024-49717-9)
Supplement: Supplementary file 1 — Supplementary Information [file 41467_2024_49717_MOESM1_ESM.pdf]

Supplementary information for: Imaging nanomagnetism and magnetic phase transitions in atomically thin CrSBr

(Dated: July 5, 2024)

CONTENTS

1. AFM to FM phase transition 2
   A. Phase boundary movement 2
   B. Formation of FM region in trilayer CrSBr 2
   C. Hysteresis loop 3

2. Temperature dependence 3
   A. Anisotropy measurement series 3
   B. Emergence of the near 2D-XY interaction model 4
   C. Magnetic field dependence of the anisotropy transition 5

3. Bulk measurements 6
   A. High-Temperature AC Magnetic Susceptibility: Phase Transition Analysis 6
   B. Low Temperature AC Magnetic Susceptibility: Bulk Phase Wall Dynamics 7

4. Micromagnetic simulations 8

5. Additional Data 8

References 17

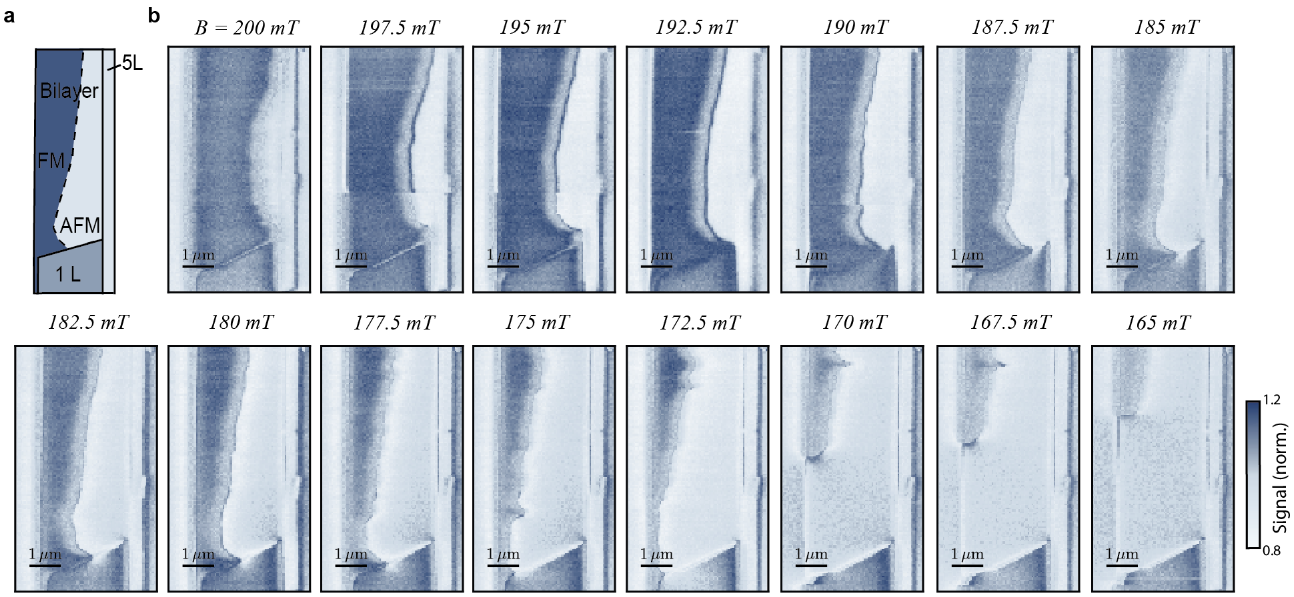

FIG. 1. Measurement series of phase boundary movement through bilayer CrSBr going from high field (200 mT) to low field (165 mT). **a** Illustration of bilayer CrSBr with FM (dark blue) and AFM (light blue) region. **b** Measurements were taken with single point Iso-B imaging.

1. AFM TO FM PHASE TRANSITION

In this section, we give additional details on the measurements of the AFM to FM phase transition in the main text. We also show additional data that was not used in the main text.

A. Phase boundary movement

As mentioned in the main text, we imaged the phase boundary movement through the bilayer using the Iso-B technique. The applied frequency was tuned to give a signal of zero while in contact away from the flake, resulting in more magnetised regions having a higher signal value. To perform these sweeps we first applied a large enough field ($B = 300$ mT) to fully initialise the bilayer into the FM state. We then decreased the field incrementally, taking an image (approximately 3 hours) at each field strength.

In Fig. 1 we show an additional dataset of the same flake but from a separate measurement series to that shown in the main text. The image region consistences of a bilayer and monolayer section with an adjoining thicker sections (4L) that remains in the AFM state throughout the series (Fig. 1 a). As the field is decreased the FM region in the bilayer recedes (Fig. 1 b). During this process, the FM phase boundary moves faster along the a-axis (intermediate axis) of the material than the b-axis (easy axis). The tendency for the phase wall to move along the a-axis was also observed in a trilayer flake which will be discussed in Section 1 B.

B. Formation of FM region in trilayer CrSBr

To investigate the domain movement in trilayer CrSBr, we imaged a trilayer section of sample $S2_{\mathrm{Encap}}$, see Fig. 2a. The small trilayer section flipped to a FM state in an external magnetic field of $B = 300$ mT applied along the NV axis, Fig. 2b. Reducing the applied field to $B = 290$ mT introduced AFM domains in the trilayer flake, Fig. 2c. These domains were evolving as a function of time: by re-imaging the flake at the same field ($B = 290$ mT) the AFM regions extended further. Reinitilaizing the trilayer flake in the FM state by applying $B = 300$ mT and subsequently imaging the flake at $B = 250$ mT shows a similar trend: AFM domains form and propagate though the flake, Fig. 2d. In both cases, the domains propagated along the a-axis (intermediate axis) of the CrSBr flake. This finding is consistent with the domain movement observed in bilayer CrSBr, Fig. 1, where propagation along the a-axis was preferred compared to the b-axis (easy axis). Differently to the domain in bilayer CrSBr, which was stable for multiple hours under an

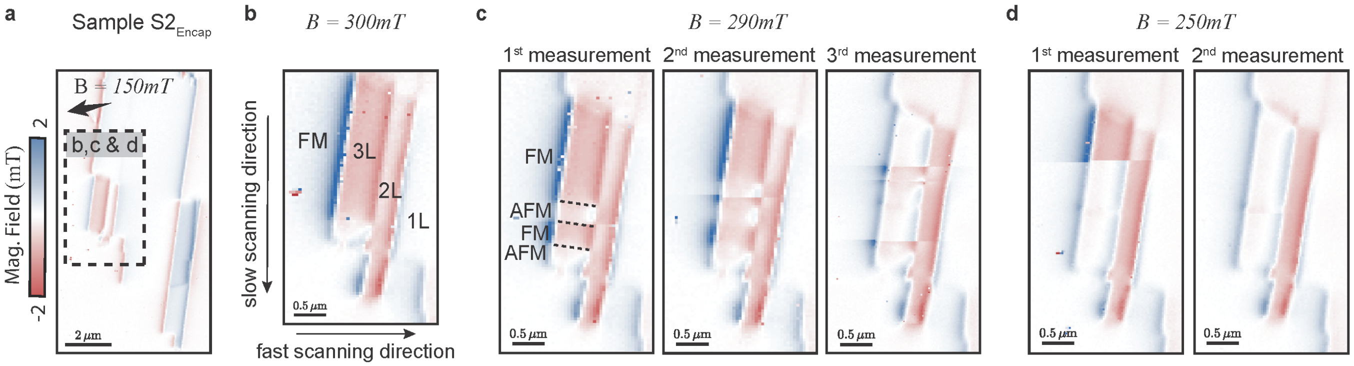

FIG. 2. Measurement series of the formation of FM/AFM regions in trilayer CrSBr. **a** Magnetic field map of sample $S2_{\text{Encap}}$. Dashed box marks the region of the trilayer region shown in panels **b-d**. **b** Magnetic field map of FM ordered trilayer CrSBr measured in an external field of $B = 300\,\text{mT}$. **c** Consecutive measurements of the formation of AFM regions in predominantly FM ordered trilayer CrSBr. The flake was first initialised in its FM state by applying $B > 300\,\text{mT}$ and subsequently measured in a lower field (290mT) for three times in a row (1st to 3rd measurement). **d** Analog to **c** but measured in a lower external B-field (250mT).

external applied magnetic field, the domain observed in trilayer was changing during scans, most likely due to a more meta-stable state of the FM/AFM phase coexistence or an effect of dragging the domain with laser light.

C. Hysteresis loop

Using the Iso-B measurement method we took a series of images at different magnetic fields to map out the hysteresis of the domain wall movement through the bi-layer, see Fig. 3**a**. These images show a clear difference in the domain wall position with different magnetic sweeping directions. In order to calculate the hysteresis of the domain wall we sum the number of pixels that are in the ferromagnetic state, as illustrated in Fig. 3**b**. Where the ferromagnetic state is define as pixels with a normalised signal greater than 1.05. This is normalised by the number of total pixels on the bi-layer region, such that

$$M = \frac{\sum \text{pixels}_{FM}}{\sum \text{pixels}}. \tag{1}$$

While is not a perfect estimation of the region it roughly approximates the FM region. The final result is then normalised by the state when the flake to purely in the FM state to force this value to be 1 and minimise issues with the imperfect threshold definition. This results in the hysteresis curve shown in Fig. 3**c**.

2. TEMPERATURE DEPENDENCE

A series of different measurements were performed at elevated temperatures with the aim of extracting the in-plane anisotropy of the material and investigating the loss of long-range order. This is discussed in detail below.

In all measurements (unless otherwise stated) the temperature was measured by a resistivity thermal couple located at the top of the piezo stack which held the sample. Due to the application of microwaves to drive the NV spin we expect some offset in the real sample temperature compared with the sensor temperature. This offset is MW power-dependent and hard to calibrate for a large range of temperatures. As such, we reference the temperature that was measured by the sensor, which ultimately means the real temperature is several unknown degrees warmer.

A. Anisotropy measurement series

To investigate the transition from AFM to FM at higher temperatures we applied a magnetic field along the intermediate a-axis of $S4_{\text{Air}}$. In doing so we partially magnetised the bilayer to point along the B-field while the monolayer was still anisotropy-dominated, resulting in it pointing along the b-axis of the material. We then maintained this magnetic field ($B = 150$ mT, $\phi = 262°$, and $\theta = 62°$) while increasing the temperature, imaging the magnetic

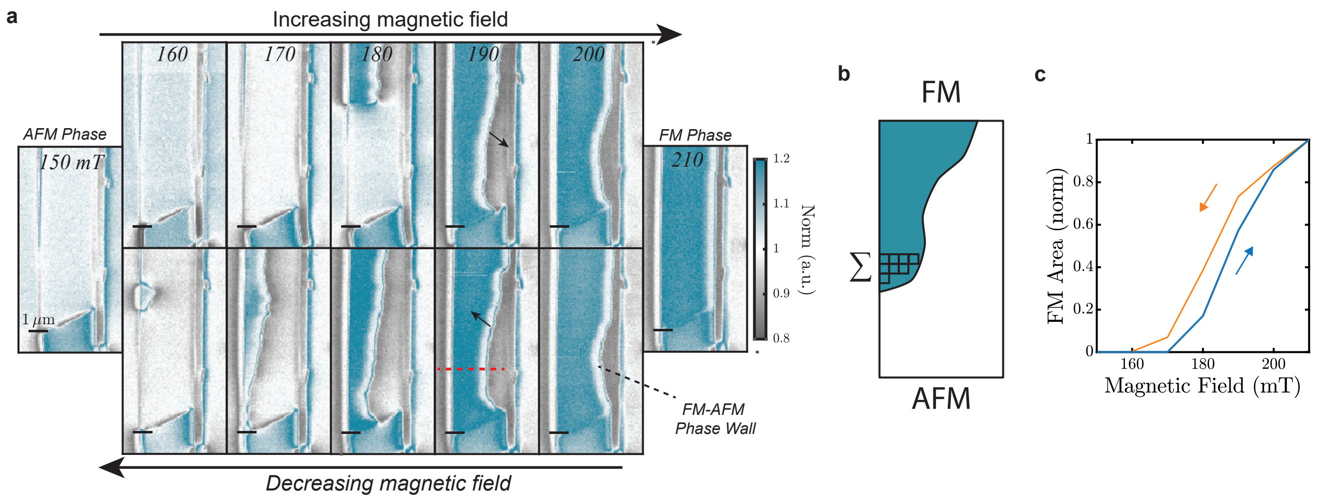

FIG. 3. Measurements of domain wall hysteresis. **a** Measurement series of the domain wall movement through the material using the Iso-B technique for both increasing and decreasing fields. **b** Illustration of partially flipped state with thresholding to sum up the pixels in the ferromagnetic state. **c** Magnetic hysteresis extracted from the images in **a**.

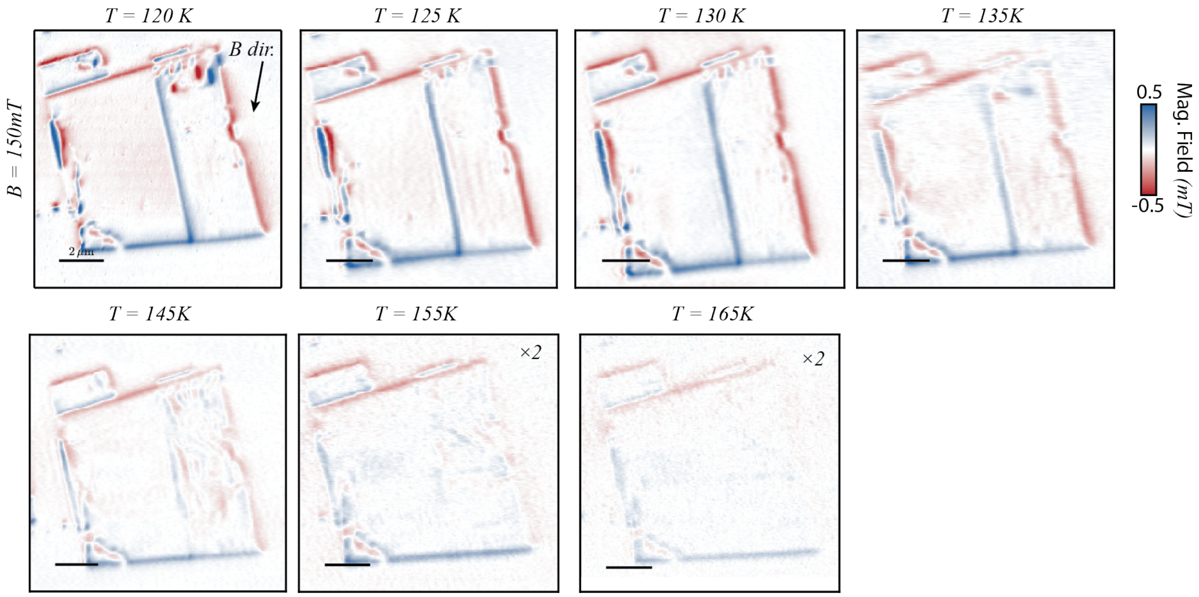

FIG. 4. Temperature series of monolayer and bilayer sample. Full temperature series of sample $S4_{\mathrm{Air}}$ shown in the main text all taken with a magnetic field strength of $B = 150mT$ roughly along the a-axis of the material with an out of plane component of approximately 28°.

field at each temperature. The magnetic images are all shown in Fig. 4. This data is used for the reconstruction of the non-uniform magnetisation and the extraction of in-plane anisotropy in the main text.

B. Emergence of the near 2D-XY interaction model

We took a series of measurements on different samples to investigate the emergence of the high temperature near 2D-XY regime, referred to here as the anisotropy transition.

Anisotropy transition in encapsulated material. In the main, text we showed magnetic images of sample $S2_{\mathrm{Encap}}$ at elevated temperatures. This measurement series was taken similar to the anisotropy series. We initialised the flake through cooling below the critical temperature with zero magnetic field. At $T = 120$ K we applied a small magnetic field $B = (\theta, \phi, |B|) = (55°, 80°, 5\mathrm{mT})$ and image the flake (Fig. 5, left panel). At this field and temperature

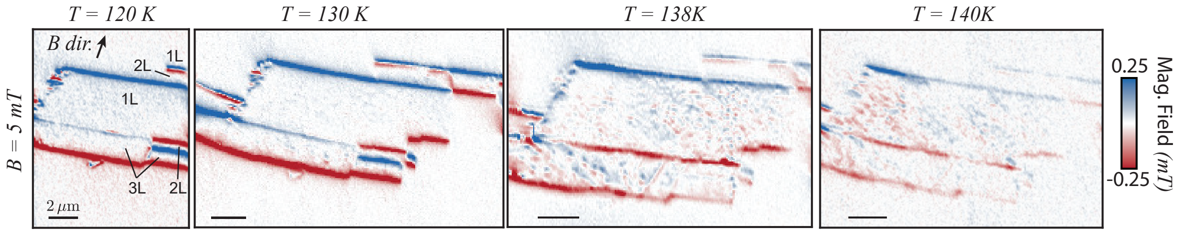

FIG. 5. Temperature series of sample D5. Full temperature series of the sample shown in the main text all taken with a magnetic field strength of $B = 5mT$ roughly along the b-axis of the material with an out-of-plane component of approximately 35°.

the odd layer (1,3) regions were FM coupled and the even-layer (2) regions were AFM coupled, resulting in zero stray magnetic field being observed. This configuration was maintained up at $T = 130$ K without evidence of deviation. However, at $T = 138$K we observed a dramatic shift, where magnetic features appear in the previously pristine mono and tri-layer regions. Increasing the temperature further to $T = 140$ K removed many of these features, illustrating the very small temperature window ($\delta T_{KT^*} \sim 10\ K$) that this effect is observable.

In this measurement series, one can observe a gradient in the magnetic field strength across the sample, particularly at higher temperatures (Fig. 5, right panels). We attribute this to a temperature gradient that is induced across the sample due to joule heating from the MW antenna, which is used to drive the NV spin and is located to the right of these images, approximately 80μm away. Additionally, during this measurement series, the temperature readout to the main sensor was lost. As such, the temperature quoted was measured further away than the rest of the measurement series. We expect that this will lead to a larger difference in the real temperature versus the measured temperature, which could be greater than 5 degrees but is unknown.

Anisotropy transition in non-encapsulated material We also performed a smaller temperature scan on Sample $S1_{\text{Air}}$, over a region containing both a bi- and tri-layer. At $T = 4K$ and low applied magnetic fields, the bilayer was observed to be perfectly AFM, with no observable stray magnetic field, while the tri-layer was uniformly magnetised over regions of 10s of microns, shown in Fig. 6**a**. When heated to near the Néel Temperature ($T = 130K \approx T_N$) the trilayer showed evidence of spontaneous reordering of the magnetisation direction. This is observed in the small red section of the left hand side in Fig. 6**b**. The change in magnetic field direction indicates that for a few lines (image taken by scanning with a faster horizontal axis and a slow vertical axis) the magnetisation direction had flipped. We also observed for a previous smaller scan at the same temperature that the magnetisation direction was opposite to the scan shown in **b**. We suggest that the system was close enough to the Néel temperature that small local perturbations in temperature may have resulted in a flipping of the magnetisation direction through heating above T_N and then cooling back down.

By increasing the temperature further to above the Néel Temperature ($T = 140$ K), we observed the bi-layer to become weakly FM, which results in a small stray field even for weak applied magnetic fields. While the tri-layer became significantly weaker in strength and formed small magnetic textures similar to the encapsulated sample (Fig. 6**c**).

C. Magnetic field dependence of the anisotropy transition

Near T_N, the anisotropy transition can be suppressed (i.e. the triaxial FM phase can be stabilized) with a modest applied magnetic field. In the case of the trilayer flake shown in Fig. 6 increasing the field to $B = 150$ mT was enough to completely remove the magnetic textures. Unfortunately, this sample was damaged before more extensive studies of these magnetic textures could be performed.

We did however take a series of different measurements on Sample $S4_{\text{Air}}$, shown in Fig. 7, taken at $T = 145K$. These measurements also confirm that magnetic field strength of $B > 200mT$ are sufficient to orientate the spins along the Zeeman direction. However, we also observe differences in the stray magnetic fields for small changes in the external magnetic field ($\Delta B < 10mT$)

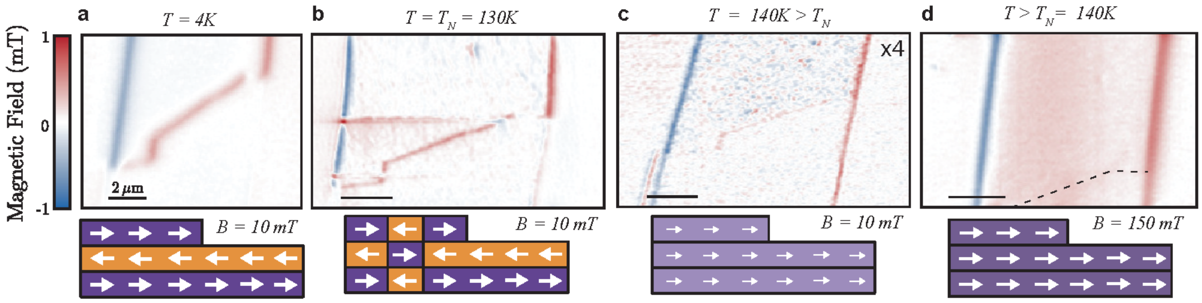

FIG. 6. Temperature series for tri- and bi-layer flake. **a** Magnetic image at $T = 120K$ with trilayer in the FM state and the bilayer in the AFM state. The rough state of the material is illustrated below the image. **b** Magnetic image at $T = 130K \approx T_n$ showing the same state as in **a** except for one region that had the opposite sign. This was concluded to be a short term flip of the entire flake rather than a local domain, see text for details. **c** Magnetic image at $T = 140K$ where both regions are in the FM state but now have zero remnant field, resulting in a reduced field strength. **d** Magnetic image at $T = 140K$ measured at a higher field $B = 150mT$ which magnetises the layers to a similar strength to before the Néel temperature. All measurements are performed with a magnetic field pointing approximately along the CrSBr b-axis with a small $\sim 30°$ out-of-plane component.

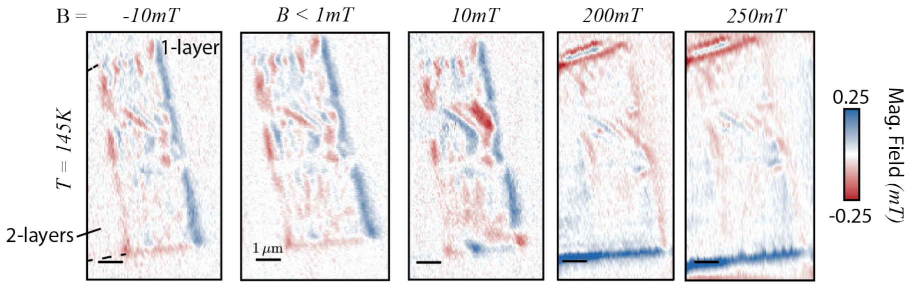

FIG. 7. Magnetic field sweep at T_c. Series of measurements of the monolayer section of sample $S4_{\mathrm{Air}}$ around the critical temperature where long-range order is disrupted.

3. BULK MEASUREMENTS

A. High-Temperature AC Magnetic Susceptibility: Phase Transition Analysis

High-temperature AC magnetic susceptibility measurements were performed to probe magnetic phase transitions near the ordering temperature. These AC magnetic susceptibility measurements were collected with the AC and DC magnetic fields oriented along the crystallographic a-axis. Measurements were collected for oscillating fields between 4 and 15 Oe; the AC susceptibility was found to be field-independent in this range, and all measurements shown here were collected with a 10 Oe oscillating field.

As described in the main text, χ_{ac} has no imaginary component for the full temperature range studied here (i.e. $\chi''_{ac} = 0$). This differs from other layered A-type antiferromagnets, such as $CrCl_3$ [1], for which AC susceptibility measurements reveal a high-temperature transition corresponding to short-range intralyer order, with features in both χ'_{ac} and χ''_{ac}, and a lower-temperature transition corresponding to interlayer antiferromagnetic order, which only generates a feature in χ'_{ac}. Despite evidence for ferromagnetic correlations in CrSBr as high as 200 K, the AC susceptibility data presented here suggests the absence of ferromagnetic domains above T_N.

The DC-field dependent measurements shown in Figure 4d were collected with an oscillation frequency of 1000 Hz. For DC fields below 2000 Oe, the AC magnetic susceptibility is unchanged as a function of the DC field. For DC fields between 2000 and 3000 Oe, a high-temperature shoulder is observed, but is not well separated from the peak at T_N. As such, only data for DC fields above 3000 Oe was considered in our analysis. Data collected for fields above 3000 Oe were then fit using a previously established procedure [2] to extract the critical exponents reported in the text. The dashed line in Figure 4d corresponds to the equation:

$$\chi_m = e^a \left(\frac{1-T}{T_m}\right)^{-\gamma} \tag{2}$$

with a = -3.25(5), T_m = 118.6(9), and γ = 2.25(5). Here, χ_m is the susceptibility value of the field-induced maximum,

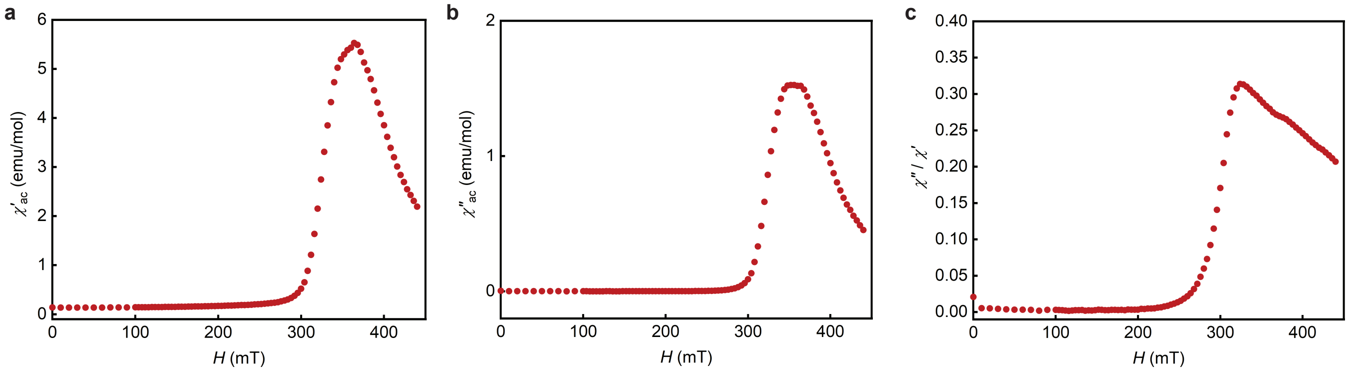

FIG. 8. AC magnetic susceptibility measurements as a function of an external magnetic field. The in-phase (**a**), out-of-phase (**b**) and the ratio (**c**) measured at $T = 2$K.

a is a constant determined by the value of the order parameter $\beta = 0.22(2)$, T_m is the temperature of the field-induced maximum, and γ is the susceptibility exponent. Notably, the value of T_m determined from these fits implies that the field-dependent features observed in AC susceptibility correspond to a zero-field ordering temperature lower than T_N; in other words, critical fluctuations above T_N suggest a lower magnetic ordering temperature than is experimentally observed. While further experiments are needed to better understand the origin of this behaviour, we speculate that the crossover from easy-plane to tricritical anisotropy may play some role in facilitating order in CrSBr, which may help to explain the inhomogeneous magnetization observed in the vicinity of T_N. We further note that this discrepancy cannot be explained purely on the basis of interlayer effects, as the magnetic ordering temperature of monolayer CrSBr is largely unchanged from that of the bulk.

B. Low Temperature AC Magnetic Susceptibility: Bulk Phase Wall Dynamics

Low-temperature AC magnetic susceptibility measurements were performed to probe domain wall dynamics in bulk crystals for the field-induced AFM-to-FM transition. Measurements were performed at 2 K with the AC and DC magnetic fields oriented along the crystallographic b-axis, with an oscillating field of 10 Oe and a drive frequency of 1000 Hz. At the bulk metamagnetic transition field (0.3 T), we observe a sharp increase in χ'_{ac} and the emergence of a non-zero χ''_{ac} (Fig. 8). We note that the magnetic field required to induce this transition in bulk crystals is approximately twice as large as that observed for the bilayer, consistent with the presence of two interlayer exchange interactions (instead of the single exchange interaction in the bilayer case). The appearance of a signal in χ''_{ac} at the phase transition indicates irreversible domain wall propagation [3], Given that the NV measurements clearly demonstrate that this phase transition is driven by phase-wall propagation through the material, this measurement supports the observation that the phase walls themselves are stable for a given external field.

In the bulk crystal AC susceptibility, we additionally observe an extended tail to high fields ($> 440\,$mT) in both χ'_{ac} and χ''_{ac}. This tail is particularly prominent in a plot of χ''_{ac}/χ'_{ac}, where a small secondary feature is observed near $\sim 380\,$mT. This secondary feature could suggest additional complexity in the spin-flip transition, such as a mix between a spin-flip and spin-flop transition, but we see no evidence for a higher-field feature in the bilayer flakes studied here.

Broadly speaking, a non-zero imaginary component in the AC magnetic susceptibility emerges when the frequency of the AC field is of similar magnitude to the characteristic frequency of a magnetic process in the sample. Under these conditions, the imaginary susceptibility is indicative of a phase lag between the sample magnetization and the AC field. As such, a non-zero imaginary susceptibility implies the presence of dissipative (irreversible) magnetic processes.

A variety of dissipative magnetic processes are known to give rise to a non-zero imaginary susceptibility, including eddy currents in metallic materials, relaxation of superparamagnets and spin glasses, and the onset of superconductivity. These processes can all be ruled out for CrSBr, which is semiconducting and shows no glassy magnetism or superconductivity.

4. MICROMAGNETIC SIMULATIONS

We employ computational micromagnetic simulations [4–6] to investigate the formation of AFM-FM phase boundaris. To do so, we simulate the fraction of the CrSBr flake that has been imaged using the scanning NV magnetometer and is shown in Fig. 2 of the main text. This implies the necessity to simulate lateral dimensions of a few micrometer. In principle, atomistic simulations would be more adequate to account for the layered structure of CrSBr, but computational limits do not allow to simulate such large structures. In our micromagnetic simulations, we mimic the layered structure of CrSBr making use of the finite difference mesh by setting the cell thickness to the thickness of one CrSBr layer. Exchange coupling between the layers is then set to be anti-ferromagnetic, while intra-layer exchange coupling is ferromagnetic.
To estimate material parameters, we consider a 200 nm x 200 nm x 200 nm simulation volume with periodic boundary conditions in all three dimensions, representing a bulk CrSBr crystal. The corresponding cell sizes are 4 nm x 4 nm x 0.8 nm, respectively. As a starting point, the inplane exchange stiffness is estimated by $A_{ex,xy} \approx k_B T_c/(2(a+b)/2) = 2.35\,\mathrm{pJ/m}$ [7] using $a = 0.350\,43\,\mathrm{nm}$, $b = 0.473\,79\,\mathrm{nm}$, and $T_c = 140\,\mathrm{K}$. The saturation magnetisation is set to its bulk value of $M_{sat} \approx 36\ \mu_\mathrm{B}/\mathrm{nm}^2$. Then, interlayer exchange coupling and strength of crystalline anisotropy axes (a and b axes of the material) are estimated in an iterative process: The relative strength of the two uniaxial crystalline easy anisotropy axes is taken from Fig. 3 **d** of the main text, and interlayer exchange coupling $A_{ex,z}$ is known to be negative and small compared to $A_{ex,xy}$. With this input these three parameters are adjusted until the bulk values for the saturation fields along the main crystal axes and the spin-flip field [8–10] are roughly matched. This leads to $K_a = 106\,098\,\mathrm{J/m^3}$, $K_b = 40\,244\,\mathrm{J/m^3}$, and $A_{ex,z} = -0.008 \cdot A_{ex,xy}$.
For the simulations of the flake we use black and white images of each layer that are deduced from optical images of the flake as geometry, as illustrated in Fig. 9 **b**. The flake is too large to be simulated as a whole, hence, we restrict ourselves to the most relevant part, which is what has been imaged experimentally. The finite element mesh is set to a size of 825 x 2525 x 6 cells with corresponding cell sizes of 4 nm x 4 nm x 0.8 nm, respectively.
An x (along b axis) gradient in $A_{ex,z}$ of about $1.25\,\mathrm{aJ/m/nm}$ is introduced to the simulation, which facilitates gradual movement of the AFM/FM phase boundary through the flake with external field. This corresponds to $A_{ex,z} = -0.008 \cdot A_{ex,xy}$ on the left hand side and $A_{ex,z} = -0.0105 \cdot A_{ex,xy}$ right hand side of the flake. Further, a small 'defect' in the top left corner of the flake, namely a spot with positive $A_{ex,z}$, serves as nucleation point for the FM domain. The saturation magnetisation is set to our measured value $M_{sat} \approx 30\ \mu_\mathrm{B}/\mathrm{nm}^2$, and $A_{ex,xy} = 1.75\,\mathrm{pJ/m}$ is adjusted in an other iterative process to match the field range of phase boundary movement, while the gradient field of $A_{ex,z}$ is kept at constant relative value. See Fig. 9 **c** for a comparison of experimental (re-scaled data from Fig. 3 **c**) and simulated hysteresis loop (x component of total magnetisation of the flake). The extremely good match of phase boundary movement field range and curve slope therein between the two data sets is owed to the before-mentioned iterative process. Note that the field range can also be matched for other values of $A_{ex,xy}$, in this case, the relative value of $A_{ex,z}$ has to be altered.

In Fig. 9 we show the gradual movement of the phase boundary through the sample in the simulated data set, which corresponds to the experimental data shown in Fig. 3. Unlike in experiment, the simulated phase boundary is perfectly vertical, except if in proximity to an edge of the sample. This is owed to the perfect orientation of the gradient in $A_{ex,z}$ with x. We have tested diagonal gradients leading to diagonal boundary orientations, implying that the irregular boundary in the experiment may originate in an equally irregular gradient in $A_{ex,z}$.

Potentially, other material parameters vary throughout the sample in a similar manner as $A_{ex,z}$. However, incorporating such a complex behaviour in our simulations is beyond what the given basis of information realistically allows.

The width of the AFM-FM domain wall is determined by the following procedure. First, we fit M_z of the linescan shown in Fig. 2 **d** (top layer) in the main text with a tanh function and extract the domain wall parameter Δ from this. Then we use the standard definition of the domain wall width $\delta_0 = \pi\Delta$, resulting in 18 nm.

5. ADDITIONAL DATA

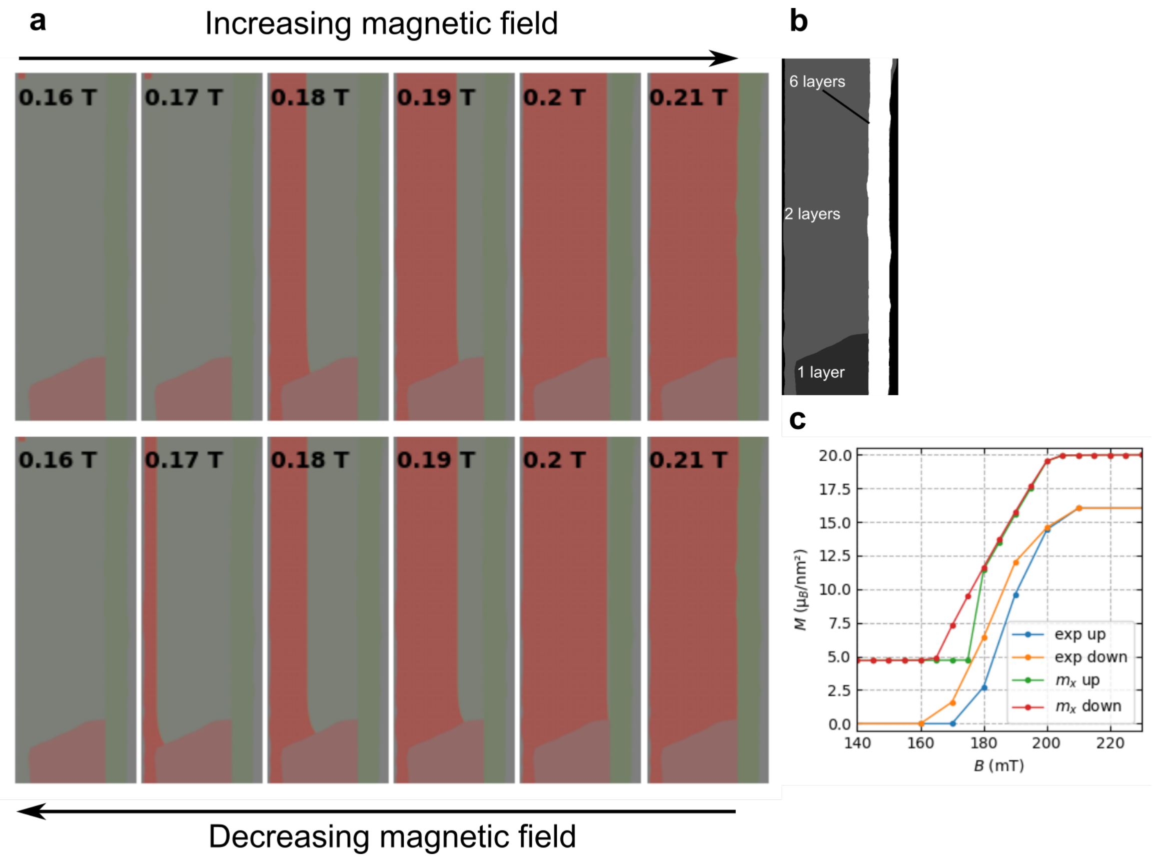

FIG. 9. Simulation of domain wall hysteresis. **a** Visualisation of magnetic states of the scanned area on the CrSBr flake. Series of the domain wall movement through the material for increasing (top) and decreasing (bottom) external magnetic field. **b** Illustration of the layer geometry **c** Hysteresis of the simulated x component of the total magnetic moment (offset for better visibility) and re-scaled experimental data

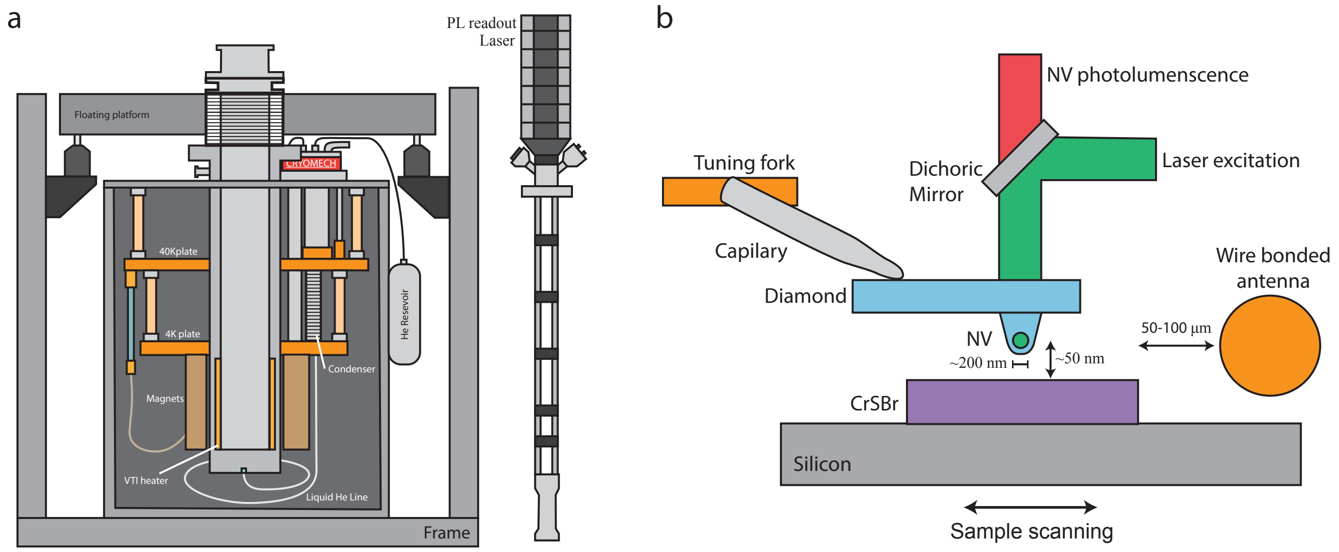

FIG. 10. Experimental principle and setup. **a** Illustration of the Atto2200 Dry system that consists of the variable temperature insert (VTI) and a standard attocube AFM-CFM microscope, with a diamond AFM cantilever (Qnami) for magnetometry. **b** Illustration of the diamond AFM that is brought into contact with the sample that is scanned to image the material. Microwave control is applied via a wire that is bonded across the sample.

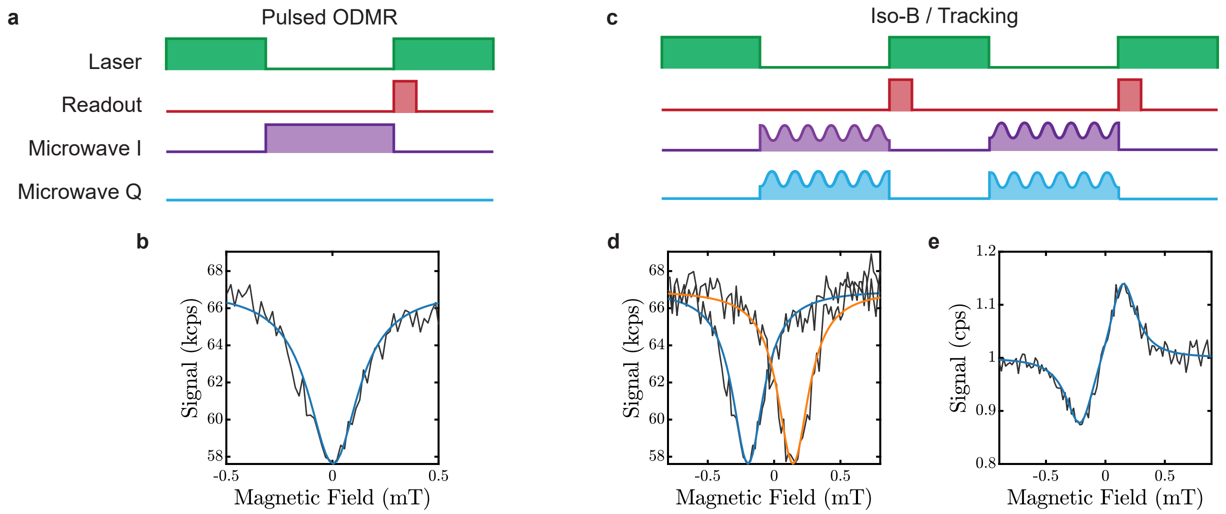

FIG. 11. **Measurement techniques.** **a** ODMR pulse sequence where the NV is initialised using a 532 nm laser pulse, followed by MW control pulse to transfer the spin into a darker state, and finally the spin is readout using another laser pulse. The continous wave version of this sequence has the laser, MW, and readout on all of the time. **b** Example ODMR spectrum (black) and Lorentzian fit (blue) taken using the sequence in **a** by sweeping the MW frequency across the resonant transition. **c** Pulse sequence for frequency modulated tracking, where an IQ-modulator is used to combine frequencies such that the applied frequency is shifted between each readout by a frequency Δf. **d** Example ODMR spectra using the frequency modulation (black) with Lorentzian fits (blue and orange). **e** Normalised signal from **d** that is used for both single point Iso-B imaging and frequency tracking where a PID loop maintains a normalised signal of 1.

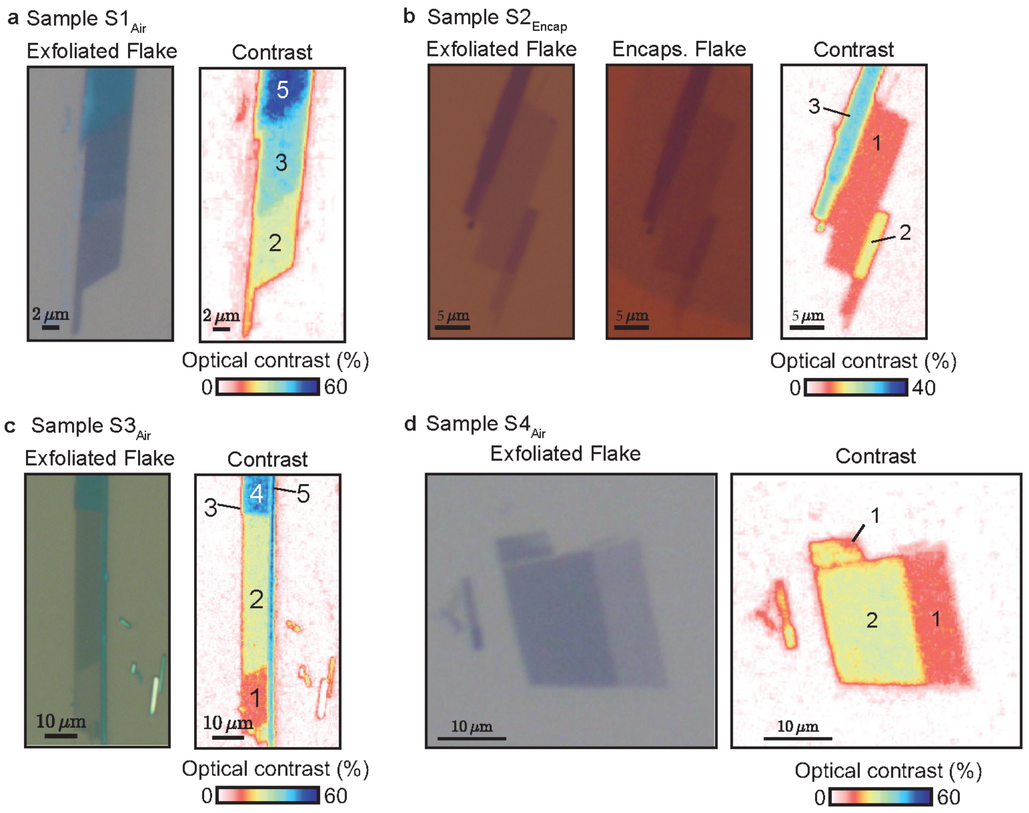

FIG. 12. Optical sample characterisation of the CrSBr crystals studied in the main text and labelled accordingly. Panels **a** to **d** show the optical microscope images of samples $S1_{\mathrm{Air}}$ to $S4_{\mathrm{Air}}$ and the extracted optical contrast used to determine the layer thicknesses. For the sample $S2_{\mathrm{Encap}}$ the encapsulated flake as depicted as well in **b**.

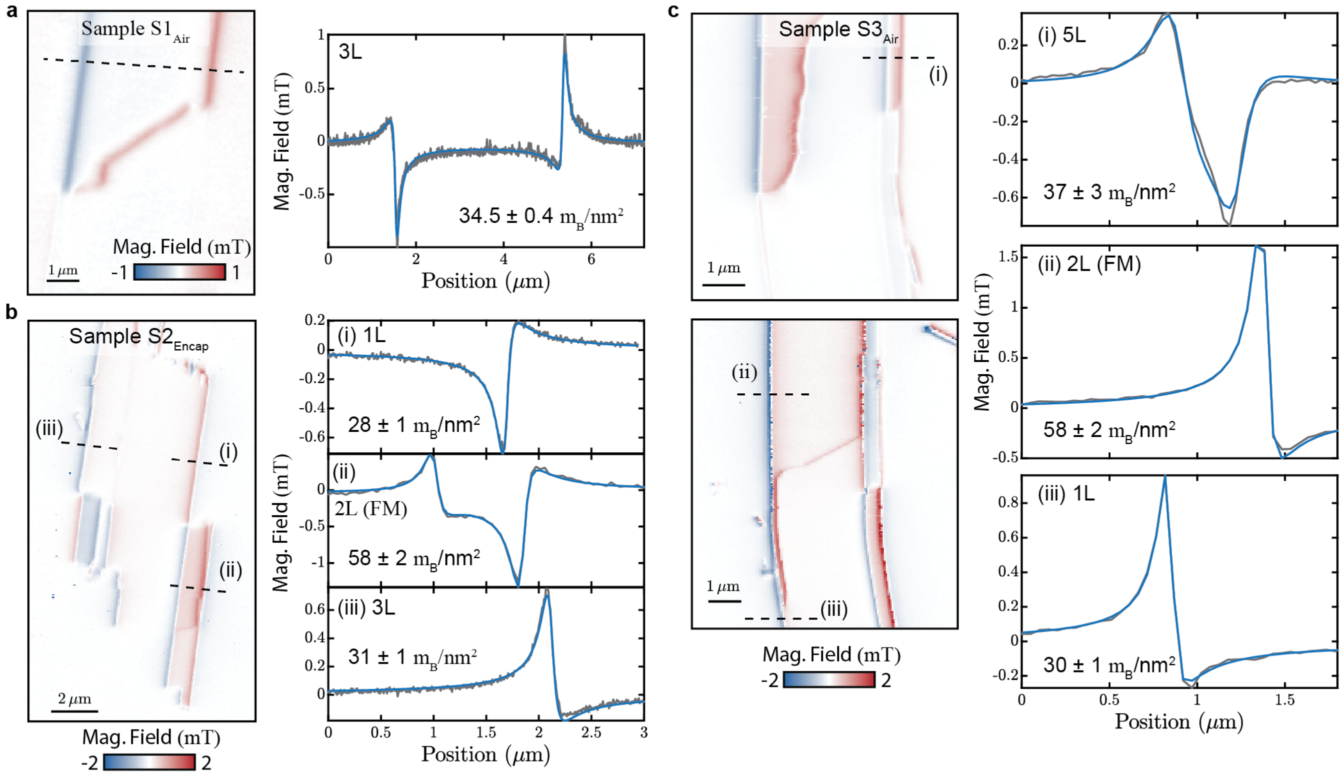

FIG. 13. Fitted Linecuts of the magnetic field for layer dependent magnetisation determination. **a** Magnetic image and linecut across the tri-layer for sample $S1_{\mathrm{Air}}$. **b** Magnetic image and linecuts from sample $S2_{\mathrm{Encap}}$ for mono, bi, and tri-layer sections. **c** Magnetic image and linecuts from sample $S3_{\mathrm{Air}}$ for mono, bi, and 5-layer sections.

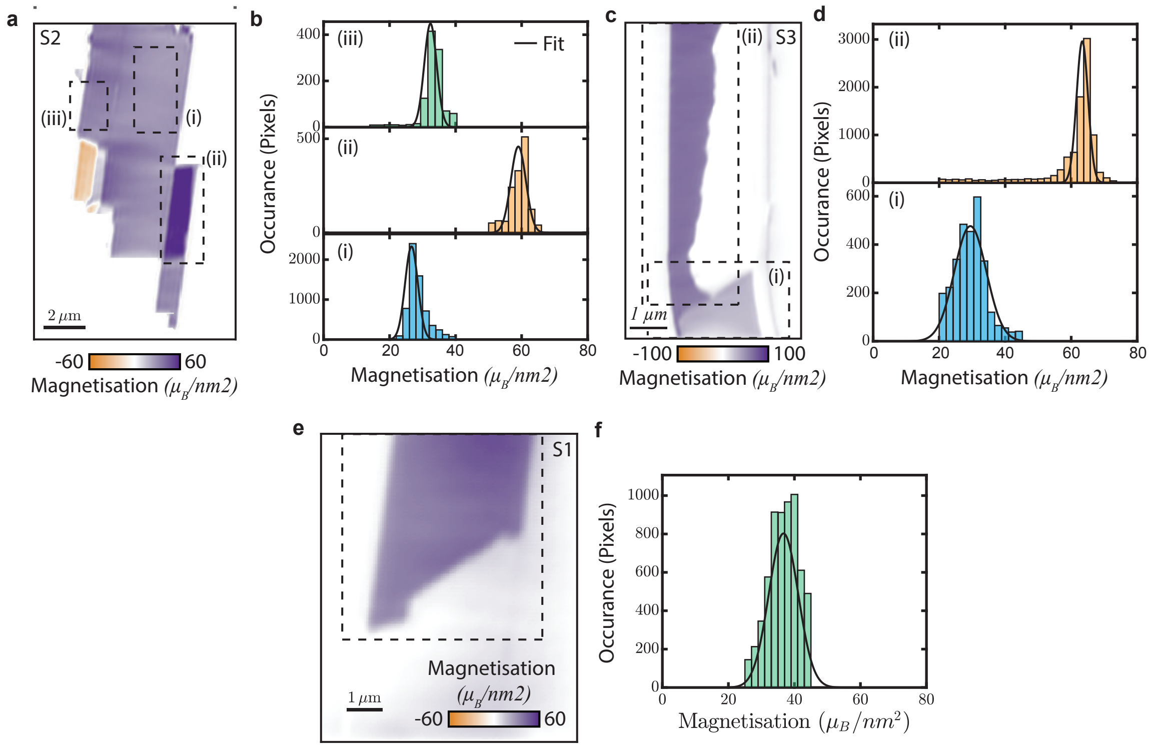

FIG. 14. Histograms of the magnetisation reconstruction for layer dependent magnetisation determination. **a** Magnetisation reconstruction for sample $S2_{\mathrm{Encap}}$ with regions for where histograms are taken. **b** Histograms from **a**, where blue is monolayer, orange is bilayer, and green is trilayer. The fit to each histogram is shown in black. **c** Magnetisation reconstruction for sample $S3_{\mathrm{Air}}$. **d** as in **b** but for the magnetisation of sample $S3_{\mathrm{Air}}$ in **c**. **e** Magnetisation reconstruction for sample $S1_{\mathrm{Air}}$ **f** as in **b** but for the magnetisation of sample $S1_{\mathrm{Air}}$ in **e**.

Layers	Sample	State	M (μ_b/nm^2)	M per layer	B (mT)	Height (nm)	Method
1	$S2_{\mathrm{Encap}}$	AFM	28 ± 1	28 ± 1	150	63 ± 1	Line cut
1	$S2_{\mathrm{Encap}}$	AFM	29 ± 2	29 ± 2	150	66 ± 4	Line cut
1	$S2_{\mathrm{Encap}}$	AFM	28 ± 3	28 ± 3	150	70	Histogram
1	$S3_{\mathrm{Air}}$	AFM	30 ± 1	30 ± 1	230	48 ± 2	Line cut
1	$S3_{\mathrm{Air}}$	AFM	27 ± 2	27 ± 2	150	60	Histogram
1	$S3_{\mathrm{Air}}$	AFM	30 ± 6	30 ± 6	230	60	Histogram
2	$S2_{\mathrm{Encap}}$	FM	58 ± 2	29 ± 1	150	70	Line cut
2	$S2_{\mathrm{Encap}}$	FM	58 ± 3	29 ± 1.5	150	70	Histogram
2	$S3_{\mathrm{Air}}$	FM	64 ± 3	32 ± 1.5	180	55 ± 2	Line cut
2	$S3_{\mathrm{Air}}$	FM	61 ± 8	30.5 ± 4	170	100	Histogram
2	$S3_{\mathrm{Air}}$	FM	63.3 ± 0.2	31.6 ± 0.1	180	60	Histogram
2	$S3_{\mathrm{Air}}$	FM	58 ± 7	29 ± 4	230	60	Histogram
2	$S3_{\mathrm{Air}}$	FM	58 ± 2	29 ± 1	230	46 ± 2	Line cut
3	$S1_{\mathrm{Air}}$	AFM	34.5 ± 0.4	34.5 ± 0.4	150	63 ± 1	Line cut
3	$S1_{\mathrm{Air}}$	AFM	36.7 ± 0.2	36.7 ± 0.2	5	60	Histogram
3	$S2_{\mathrm{Encap}}$	AFM	31 ± 0.7	31 ± 0.7	150	69 ± 2	Line cut
3	$S2_{\mathrm{Encap}}$	AFM	33 ± 3	33 ± 3	150	70	Histogram
3	$S2_{\mathrm{Encap}}$	FM	98 ± 5	33 ± 2	300	73 ± 5	Line cut
5	$S3_{\mathrm{Air}}$	AFM	37 ± 3	37 ± 3	172.5	111 ± 10	Line cut
5	$S3_{\mathrm{Air}}$	AFM	38 ± 3	38 ± 3	170	100	Histogram
5	$S3_{\mathrm{Air}}$	AFM	37 ± 2	37 ± 2	160	100	Histogram

TABLE I. Measurements of the magnetisation of different layer thicknesses at a temperature of approximately 4 K. Blue color highlights data points used in Fig.1 e of the main text. Note: we did not acquire any data at low temperature of $S4_{\mathrm{Air}}$ that was suitable for extracting the magnetisation.

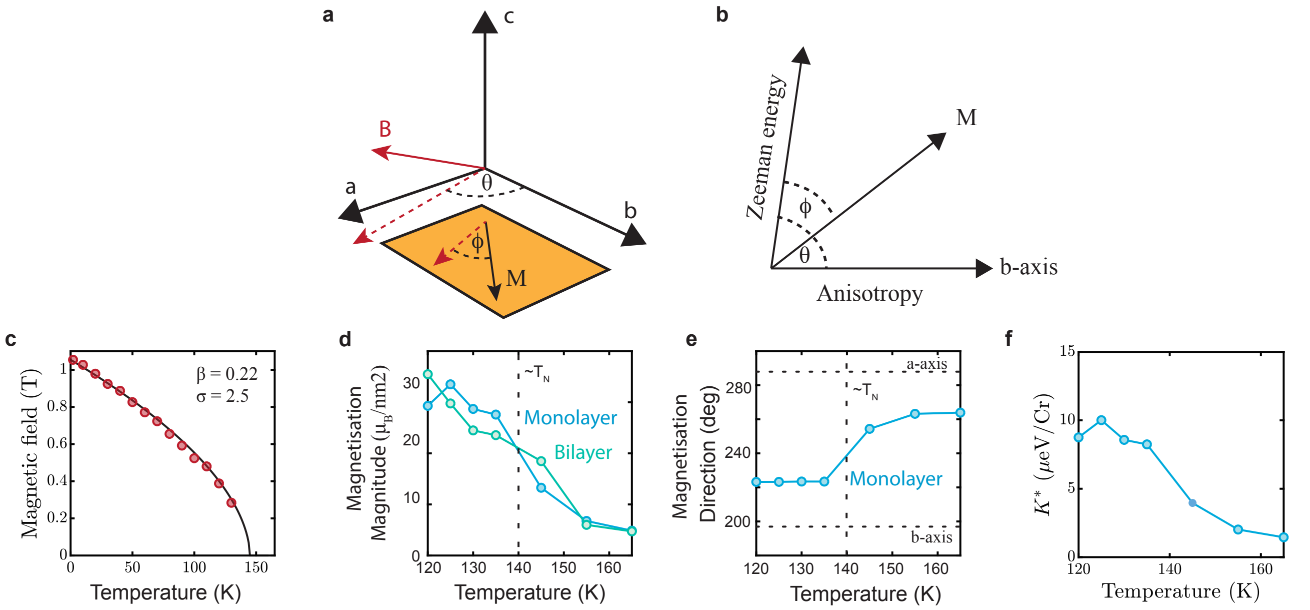

FIG. 15. Method for extracting anisotropy from NV measurements. **a** Illustration of a magnetic flake with an easy axis along the b-axis and a magnetic field at an arbitrary angle (red arrow, dashed for projection onto the a-b plane). **b** Illustration of the energy landscape for the the magnetisation, where the magnetisation points along a direction that is given by the combination of Zeeman and anisotropy energy. **c** Bulk measurement of the flipping field along the a-direction and extracted critical behaviour. **d,e** Magnetisation strength (**d**) and direction (**e**) dependence as a function of temperature extracted from the measurement series discussed in Sec. **??**. **f** Calculated in-plane anisotropy as a function of temperature (blue) and the theoretical prediction from the Stoner-Wohlfarth model (black).

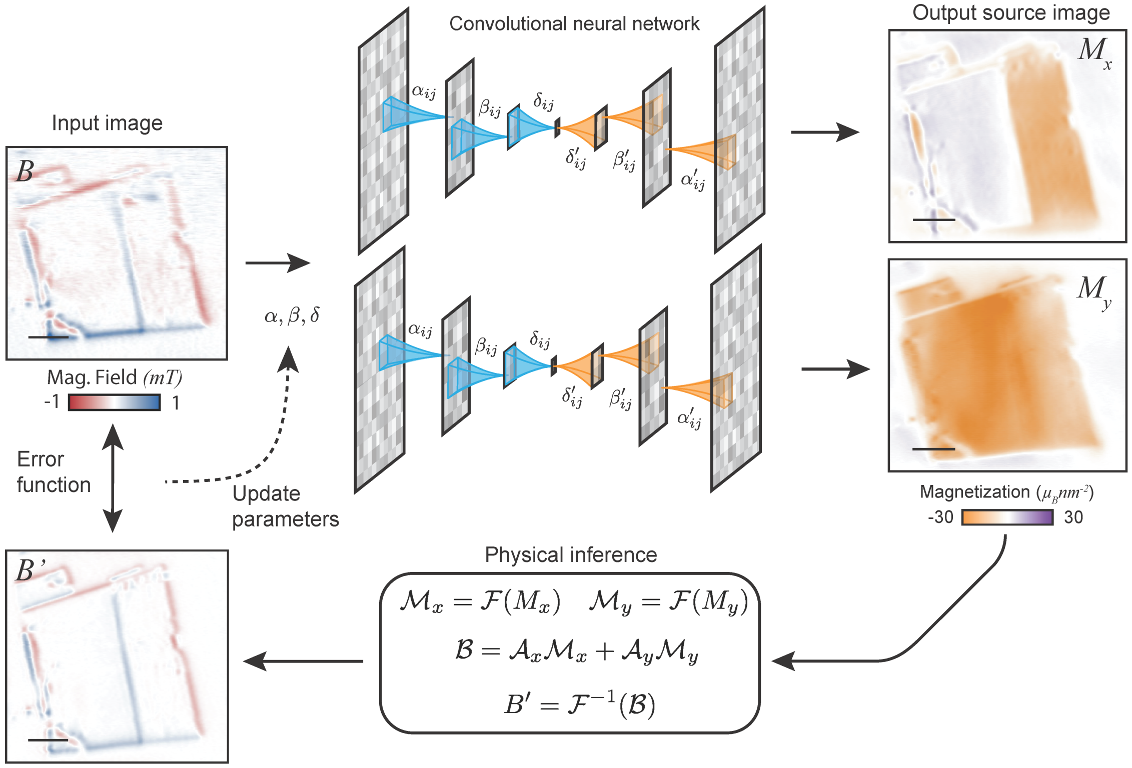

FIG. 16. Illustration of the neural network reconstruction of non-uniform direction magnetisation. The input magnetic field B is fed into two neural networks to produce both a M_x and M_y image, which is fed back to reconstruct a magnetic field image to produce an error function. The scale bar is 2 mum

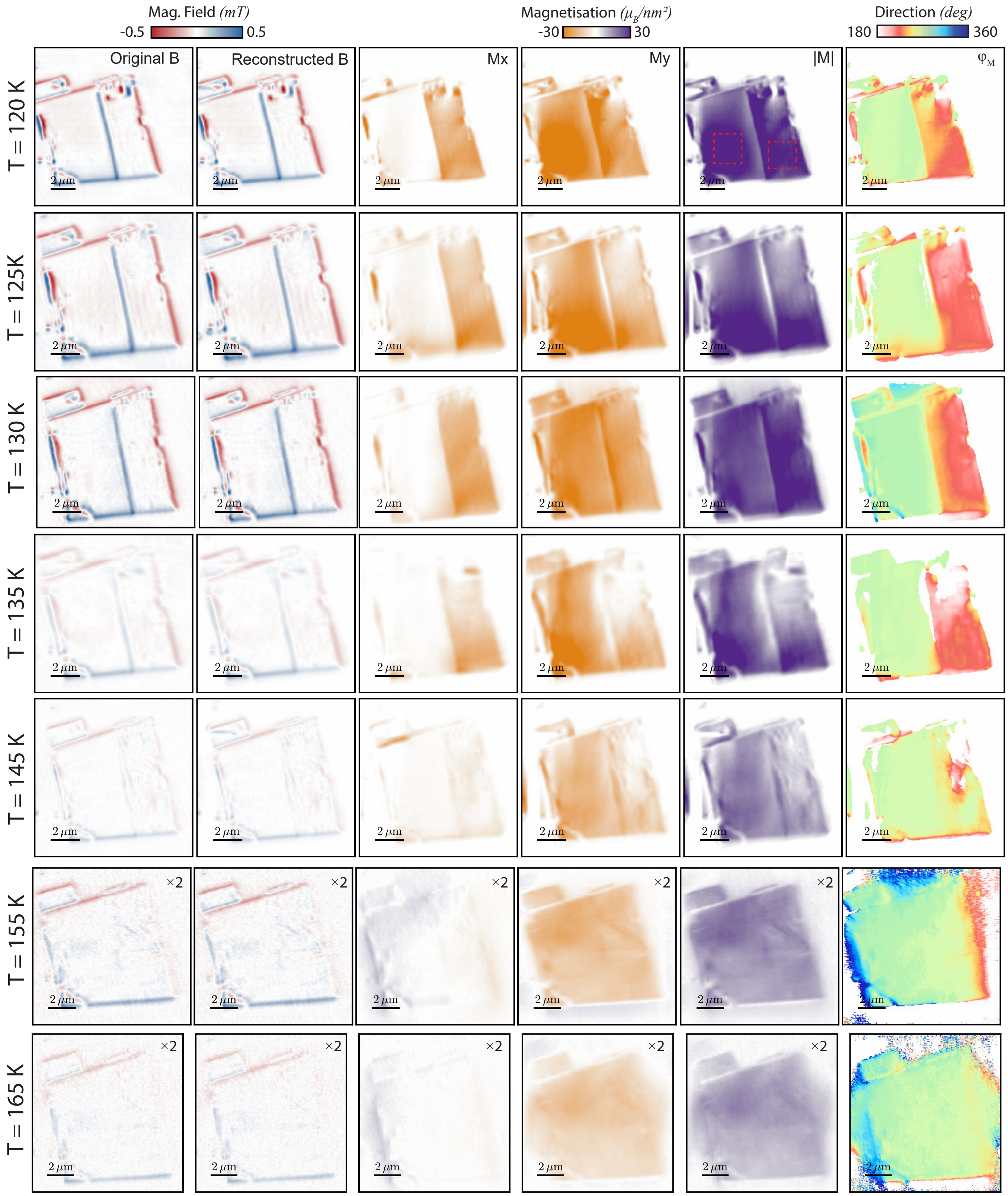

FIG. 17. Magnetisation reconstruction of all the magnetic images taken at different temperatures in the main text. The rough area for the average magnetisation magnitude and direction is shown as black dashed squares on the top row. The panels from left to right are: The original magnetic field, the reconstructed magnetic field by the neural network, the magnetisation along the X-direction of the image, the magnetisation along the Y-direction of the image, the magnitude of the magnetisation, and the direction of the magnetisation. The bottom two rows have the data multiplied by two to make the data visible. The magnetisation direction has had a threshold-based background subtraction to remove magnetisation being added to regions where no magnetic material was present.

[1] Y. Liu and C. Petrovic, Physical Review B **102**, 014424 (2020).
[2] J. H. Zhao, H. P. Kunkel, X. Z. Zhou, G. Williams, and M. A. Subramanian, Physical Review Letters **83**, 219 (1999).
[3] M. Bałanda, Acta Physica Polonica A **124**, 964 (2013).
[4] A. Vansteenkiste, J. Leliaert, M. Dvornik, M. Helsen, F. Garcia-Sanchez, and B. Van Waeyenberge, AIP Advances **4**, 107133 (2014).
[5] J. Leliaert, J. Mulkers, J. De Clercq, A. Coene, M. Dvornik, and B. Van Waeyenberge, AIP Advances **7**, 125010 (2017).
[6] L. Exl, S. Bance, F. Reichel, T. Schrefl, H. Peter Stimming, and N. J. Mauser, Journal of Applied Physics **115**, 17D118 (2014).
[7] J. M. D. Coey, *Magnetism and Magnetic Materials* (Cambridge University Press, 2010).
[8] C. Boix-Constant, S. Mañas-Valero, A. M. Ruiz, A. Rybakov, K. A. Konieczny, S. Pillet, J. J. Baldoví, and E. Coronado, Advanced Materials **34**, 2204940 (2022).
[9] E. J. Telford, A. H. Dismukes, R. L. Dudley, R. A. Wiscons, K. Lee, D. G. Chica, M. E. Ziebel, M.-G. Han, J. Yu, S. Shabani, A. Scheie, K. Watanabe, T. Taniguchi, D. Xiao, Y. Zhu, A. N. Pasupathy, C. Nuckolls, X. Zhu, C. R. Dean, and X. Roy, Nature Materials **21**, 754 (2022).
[10] S. A. López-Paz, Z. Guguchia, V. Y. Pomjakushin, C. Witteveen, A. Cervellino, H. Luetkens, N. Casati, A. F. Morpurgo, and F. O. von Rohr, Nature Communications **13**, 10.1038/s41467-022-32290-4 (2022).
